# DHAFormer: Dual-channel hybrid attention network with transformer for polyp segmentation

**DOI:** 10.1371/journal.pone.0306596

**Published:** 2024-07-10

**Authors:** Xuejie Huang, Liejun Wang, Shaochen Jiang, Lianghui Xu

**Affiliations:** School of Computer Science and Technology, Xinjiang University, Urumqi, China; National Chengchi University, TAIWAN

## Abstract

The accurate early diagnosis of colorectal cancer significantly relies on the precise segmentation of polyps in medical images. Current convolution-based and transformer-based segmentation methods show promise but still struggle with the varied sizes and shapes of polyps and the often low contrast between polyps and their background. This research introduces an innovative approach to confronting the aforementioned challenges by proposing a Dual-Channel Hybrid Attention Network with Transformer (DHAFormer). Our proposed framework features a multi-scale channel fusion module, which excels at recognizing polyps across a spectrum of sizes and shapes. Additionally, the framework’s dual-channel hybrid attention mechanism is innovatively conceived to reduce background interference and improve the foreground representation of polyp features by integrating local and global information. The DHAFormer demonstrates significant improvements in the task of polyp segmentation compared to currently established methodologies.

## Introduction

Colorectal cancer (CRC) is a prevalent and lethal form of cancer worldwide, responsible for over 694,000 deaths annually. It ranks high in terms of cancer incidence and mortality rates, posing a substantial risk to public health [1]. The prevailing medical consensus holds that CRC typically develops from adenomatous polyps through several stages. Early screening and removal of colon polyps can reduce the risk of CRC [2, 3]. Effective early screening and preventive strategies are critical in reducing the incidence and mortality rates associated with CRC. Nonetheless, the variability in polyp sizes and shapes, inconsistent image quality, and the presence of indistinct features in medical imaging complicate the accuracy of colonoscopy procedures. This introduces difficulties and risks in CRC screening and prevention.

The development of artificial intelligence has spurred research into learning algorithms for computer-aided diagnostic (CAD) systems, aiming to detect and delineate polyps autonomously. This advancement could improve physicians’ capabilities in identifying lesions and reducing missed detection rates [4–6]. However, polyp segmentation, crucial for enhancing the efficiency and quality of colonoscopy, encounters numerous technical challenges. The difficulty in differentiating polyps from their surrounding mucosa during colonoscopy is often attributed to their similar color, texture, and shape, particularly under variable illumination and in situations involving flat lesions or inadequate bowel preparation.

With the advent of deep learning, convolutional neural networks (CNNs) have established the foundation for contemporary polyp segmentation methods. Full convolutional network (FCN) [7] was initially suggested for semantic segmentation, and later on, their variations [8, 9] also made great strides in polyp segmentation tasks. Most segmentation models employ an encoder-decoder structure based on the UNet [10] architecture, typically built from a convolutional layer. Despite the dominance of UNet in polyp segmentation, it and its subsequent variants [11–13] face a similar problem as the CNN model: a lack of modeling ability for global correlations. This limitation primarily stems from the fact that CNNs only extract local information and cannot effectively capture global correlations.

In computer vision, researchers are exploring the use of the Transformer [14] architecture, known for utilizing self-attention mechanisms to establish connections among distant elements in input data. The Vision Transformer (ViT) [15] adapts this architecture for image recognition tasks by dividing an image into patches and processing them as a sequence, which reduces computational costs and enhances the processing of large images. ViT has been proven effective in various image segmentation tasks. Recent studies, such as those involving Polyp-PVT [16], SSFormer [17], FCBFormer [18] and DuAT [19] indicate that Transformer-based models achieve exceptional performance in polyp segmentation tasks. However, despite their enhanced accuracy in segmentation, these models often struggle with indistinct polyp boundaries. This is partly due to the small scale of existing polyp datasets, which do not represent the full range of polyp sizes and can lead to pixel imbalance due to the low proportion of polyp pixels in the overall image. Another challenge is the shape of polyps. Their irregular and jagged contours can make it difficult for networks to identify edge pixels accurately. Classical networks may have limitations in effectively segmenting polyps of various sizes.

Based on the aforementioned factors, we offer a novel method called DHAFormer, which features a dual-channel hybrid attention (DHA) module, enhancing the model’s capacity for foreground perception of polyps alongside global and local information processing. Crucially, the DHAFormer incorporates a multi-scale channel fusion module (MCFM) designed to aggregate features across multiple scales, bolstering the detection and delineation of polyps of varying sizes. The MCFM functions by emphasizing salient features while diminishing less relevant ones, thereby sharpening the polyp’s visibility and segmentation accuracy. It integrates a channel attention mechanism that assigns adaptive weights to each channel, providing a nuanced feature representation based on the polyp’s contextual surroundings. This multi-faceted approach allows for more precise and detailed analysis, substantially improving the performance of polyp segmentation.

Our significant contributions are as follows:

1. We design the MCFM, which integrates multi-scale feature extraction with a channel attention mechanism to optimize local detail perception and enhance sensitivity to polyps of various sizes.
2. We propose a DHA module that combines global and local features, thereby enhancing the model’s sensitivity to information and improving its ability to recognize foreground information effectively.
3. We validated the DHAFormer through comprehensive experimentation on two prestigious public datasets, and the results demonstrated the effectiveness of our proposed model.

## Related work

### Convolutional neural networks

CNN is a cornerstone of deep learning in medical imaging, excelling in tasks like target detection [20], classification [21], and semantic segmentation [22]. UNet [10] stands out for its effective spatial hierarchy management and localization precision in image segmentation. UNet++ [11] evolved from UNet [10] by introducing nested skip pathways and deep supervision for improved feature propagation and segmentation accuracy. UNet3+ [12] employs a comprehensive jump-connected structure for detailed information extraction and deep supervision for improved feature representation in polyp segmentation. UACANet [3] and DCRNet [23] explored the region of uncertainty and the relationships within and across image contexts, respectively. Jain et al. [24] conducted a comparative study of deep learning-based segmentation models, demonstrating the effectiveness of UNet and SegNet architectures using MobileNetV1 for polyp localization in wireless capsule endoscopy(WCE) images. WCENet [25] featuring a two-phase process that classifies WCE images into four categories and uses an attention-based CNN with a SegNet-based localization framework. Jain et al. [26] propose a CNN with meta-feature learning for wireless capsule endoscopy image classification, which handles intra-class variability and proficiently categorizes gastrointestinal images as either normal or abnormal. Despite their proficiency in local feature extraction, convolutional operations are limited in their ability to capture global image information. Comparatively, global information is crucial to separating foreground from background in polyp segmentation. Therefore, relying on more than convolutional operations may lead to poor-quality segmentation.

### Attention mechanism

To improve the feature representation capabilities of CNNs, some researchers have recently introduced attention methods. Attention methods enable networks to prioritize salient aspects of the input data. For example, AG-Sononet [13] created an attention gate module that permits the network to concentrate on important information while preserving computational efficiency. To enhance UNet++ [11] for polyp segmentation, AG-ResUNet++ [27] combines attention gates with the ResNet [28] foundation. The reverse attention module used by PraNet [29] forces focus on the line separating a polyp from its surroundings. CoInNet [30] proposes a novel concern mechanism with convolution, involution, and statistical feature concern units for polyp segmentation. Huang et al. [31] proposed a polyp segmentation network using a hybrid channel-spatial attention and pyramid global context guided feature fusion, achieving significant improvements in segmentation accuracy across multiple datasets. Overall, the attention module can bring performance gains to most CNNs and neural networks. Nonetheless, even with attention enhancements, CNNs face difficulties in capturing the extensive spatial relationships between distant input segments.

### Vision transformer

Transformer [14] has revolutionized the field of natural language processing with its ability to capture long-range dependencies in input sequences through self-attention mechanisms. Its application has expanded to medical imaging tasks such as polyp segmentation, demonstrating its versatility. For example, Transfuse [32] employs a dual-branch structure combining Transformer and CNN to leverage both global and local feature extraction. Polyp-PVT [16] integrates a pyramid vision transformer to enhance feature robustness. Segtran [33] proposes a compressed attention block to normalize self-attention and extend blocks to learn diversified representations. SSFormer [17] proposes an aggregate of local and global features stepwise, improving the model’s processing ability. USegTransformer-P and USegTransformer-S [34] integrate transformer-based and convolution-based encoders to enhance precision in medical image segmentation tasks, combining local and global features effectively. Recent progress in transformer-based medical image analysis [35] explores the adoption of transformers in medical image analysis (MIA), highlighting their utility in improving classification, segmentation, and other MIA tasks through their ability to handle complex data and enhance feature extraction. WDFF-Net [36] proposed scale-sensing feature fusion to solve the problem of large changes in polyp size and shape. Wang et al. [37] propose a new architecture for polyp segmentation that uses CNN and transformers as encoders to capture local information and remote dependencies. These models showcase improved handling of polyp boundaries and feature robustness but still face challenges with irregular polyp shapes.

DHAFormer differentiates itself from other methods by integrating a MCFM and a DHA mechanism, which together enhance the segmentation accuracy and robustness by effectively capturing both local and global features.

## Methodology

### Overall DHAFormer

**Fig 1:**
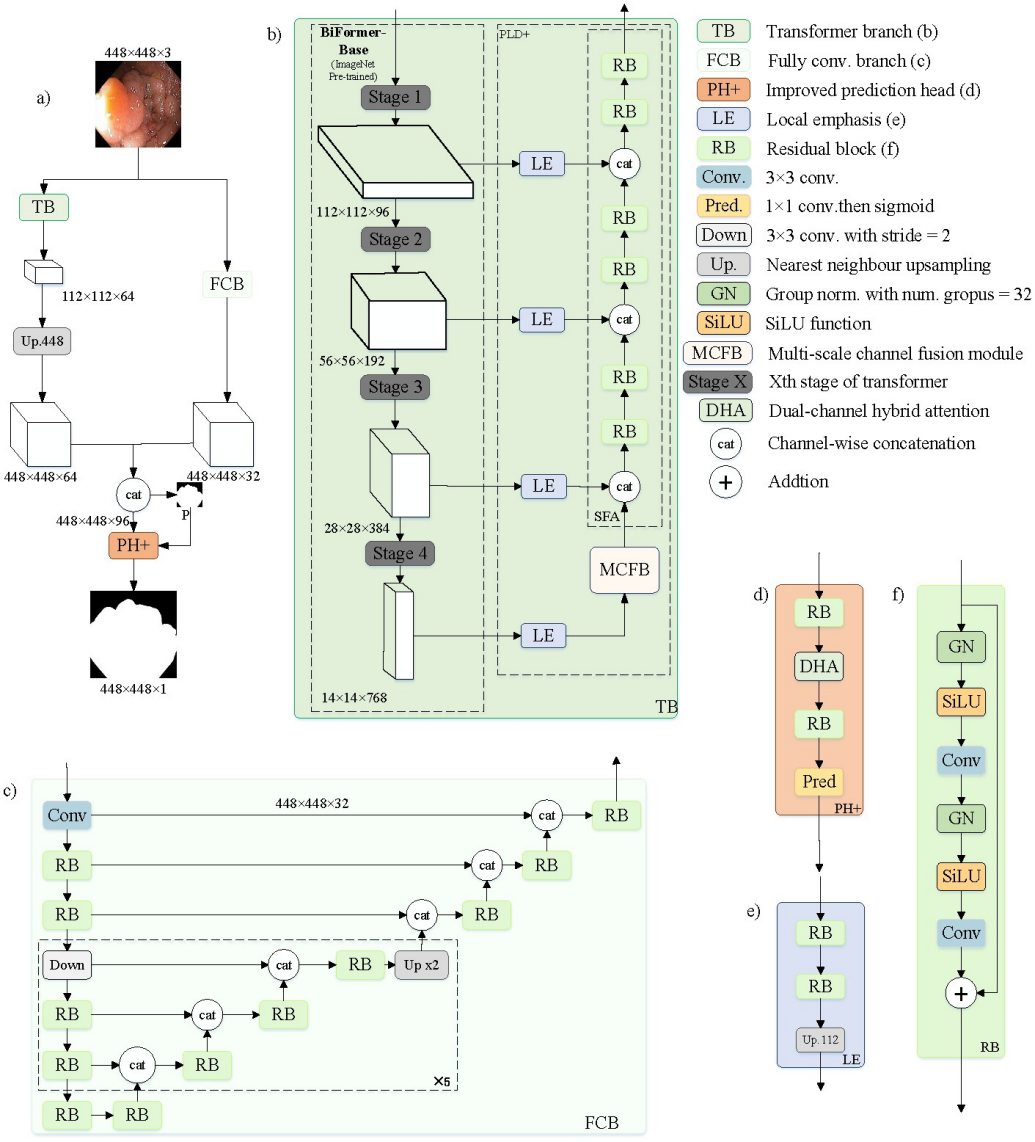
Overall network architecture. (a) DHAFormer. (b) Transformer branch. (c) Fully convolutional branch. (d) Improved prediction head module. (e) Local emphasis module. (f) Residual block.

Fig 1a illustrates the network’s general design, which uses two parallel branches: the FCN branch (FCB) and the transformer branch (TB). The FCB is mainly used to output the full-size feature maps for extracting local information. We use the BiFormer [38] architecture in the TB branch as an encoder. The TB branch outputs reduced-size semantic feature maps, focusing on relevant regions through an MCFM for extracting global information and then up-sampling to full-size features. The improved prediction head (PH+) will then process the combined result features of the two branches. To better focus on the foreground polyp region and capture global dependencies at various scales, we designed a DHA module for the PH+ module. This enables the model to identify and segment the polyp region more accurately. The FCB is aligned with FCBFormer [18].

### Fully convolutional branch (FCB)

The overall structure of FCB is shown in Fig 1c. We adopted the same parameters for the FCB of our network as FCBFormer [18], which permits the fusion of multiscale features and, when combined, features extracted from the transformer branch enables more precise prediction of full-size segmentation maps.

### Transformer branch (TB)

#### Transformer encoder

In this study, unlike the approach in FCBFormer [18], an ImageNet pre-trained BiFormer [38] serves as the image encoder within the TB framework, substituting the previously used pyramid vision transformer v2 (PVTv2) [39]. The selected BiFormer model is the ‘base’ variant, boasting 56.8 million parameters and leveraging bi-level routing attention. This method facilitates dynamic, per-query sparse attention, allowing for an enhanced focus on pertinent key regions. The implementation of FCBFormer we used in our experiment uses BiFormer [38] as the encoder for the TB.

The overall architecture of the transformer encoder is shown in Fig 1b. we obtain four distinct feature pyramid levels(El, E2, E3, E4), ranging from coarse to fine, via the BiFormer encoder. El, E2, and E3 are categorized as low-level features, amalgamate detailed feature data with some degree of noise and irrelevant details. By enhancing and examining these traits, they can offer fine-grained details to enhance advanced features. As part of the advanced decoder input, E4 is an advanced feature that allows for exact target area location.

#### Transformer decoder

Similar to FCBFormer [18] settings, the transformer encoder returns features with four levels, which we use as inputs to an improved progressive locality decoder (PLD+) to obtain multiple scales of features. The PLD+ consists of four Local Emphasis (LE) modules, an MFCM, and a stepwise feature aggregation (SFA) module, each LE module dealing with features at one level of the feature pyramid. The role of the LE modules is to enhance the local features in the feature representation, as the transformer-based model is relatively weak in this respect. After the fourth layer, features are processed by LE module, MCFM is added to enhance the processing of high-level features by the network. We then fuse the outputs of the three LE modules and an MCFM into a multi-scale feature map for predicting polyp regions in the image via the SFA module. Compared to the traditional transformer structure, this alternative can more effectively utilize local features in the image to enhance segmentation.

#### Multi-scale channel fusion module (MCFM)

Generally, polyps vary widely in size and shape, so a segmentation method that can adapt to different scales is needed. We propose a multi-scale channel fusion module, which combines multi-scale features with channel attention to deal with multi-scale problems effectively. Precisely, we first extract multi-scale features using different convolution branches, then compute channel attention to adapt the features of different channels, and finally combine multi-scale features and channel attention. In this way, the model can be better adapted to objects and features at different scales.

**Fig 2:**
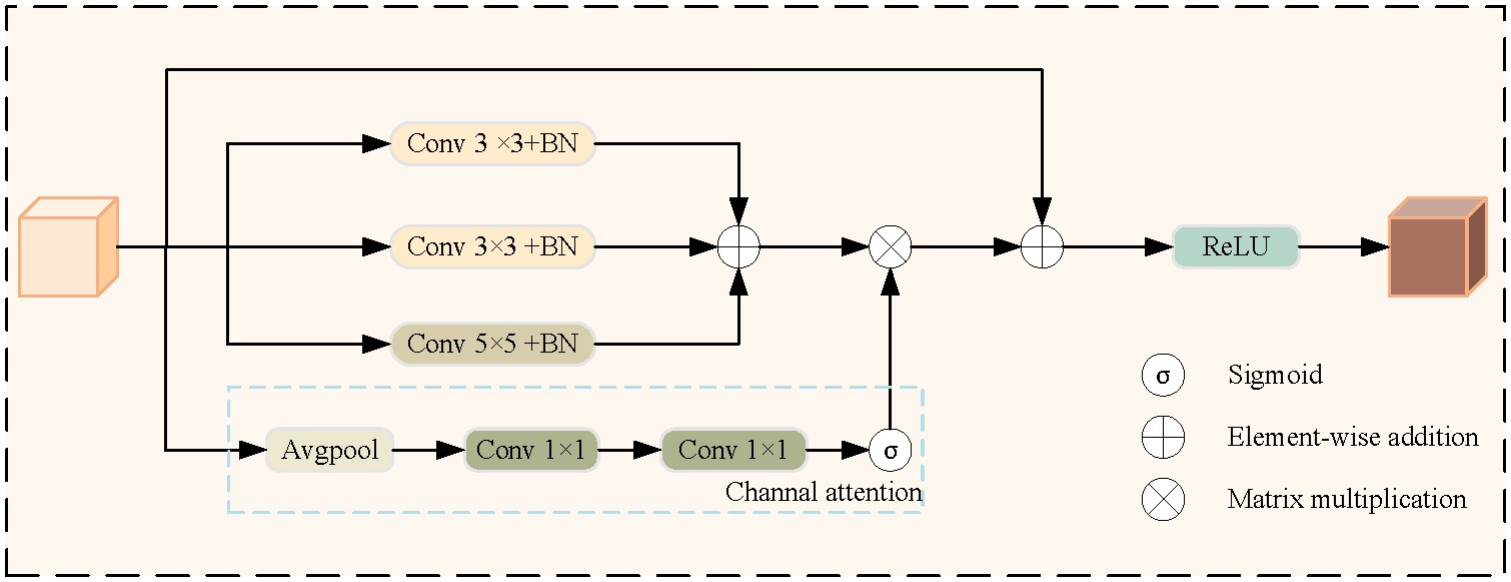
Overall architecture of MFCM module.

Fig 2 shows the detailed design of MCFM. The input of MCFM is $E_{4}^{\prime }\in R^{64\times \frac{H}{4}\times \frac{W}{4}\;}$, which is $\;E_{4}\in R^{768\times \frac{H}{32}\times \frac{W}{32}\;}$ after being processed by LE. Firstly, multi-scale feature extraction is carried out to capture information of different scales. Each branch performs a convolution operation, where branches 1 and 2 use a 3x3 convolution kernel, and branch 3 uses a 5x5 convolution kernel. These operations extract feature details to accommodate polyps of different sizes and shapes. The features obtained through these three branches are then added together. This operation can be expressed as:

$$\begin{array}{c} F_{i}=BN\left(Conv_{3\times 3}\left(E_{4}^{\prime }\right)\right),\left(i=1,2\right) \end{array}$$ (1)

$$\begin{array}{c} F_{3}=BN\left(Conv_{5\times 5}\left(E_{4}^{\prime }\right)\right) \end{array}$$ (2)

$$\begin{array}{c} F^{\prime }=F_{1}⊕F_{2}⊕F_{3} \end{array}$$ (3)

where BN indicates the BatchNorm [40] operation. ⊕ denotes element-wise addition.

Next, the channel attention mechanism calculates the channel attention weight through two convolutional layers. This weight is used to adaptively weight the features of different channels to determine which channels are most critical for the segmentation task. The channel attention is calculated by a Sigmoid [41] activation function and two convolution operations in the formula. The formula is as follows:

$$\begin{array}{c} W=\sigma (Conv_{1\;\;\;\times \;\;\;1}\left(Conv_{1\;\;\;\times \;\;\;1}\left(Avg\left(E_{4}\right)\right)\right)) \end{array}$$ (4)

where *σ* stands for the Sigmoid [41] function, which converts the convolutional output to a weight between 0 and 1. *Conv*_1×1_ is a 1x1 convolution operation used to change the dimension of the feature channel. *Avg* stands for adaptive average pooling operation, and it reduces the spatial dimension to facilitate global computation of channel attention.

In the multi-scale feature fusion stage, features from three branches are combined to integrate information at different scales. This helps the module better adapt to multi-scale objects, no matter how size changes. *F*_*weight*_ is by adding features *F*′ and channel weight *W* is achieved by multiplying elements by elements:

$$\begin{array}{c} F_{weight}=F^{\prime }⊗W \end{array}$$ (5)

Finally, we combine the weighted features with the original input features by learning the parameter *α* and generating the *F*_*out*_ by the *ReLU* activation function:

$$\begin{array}{c} F_{out}=ReLU\left(\alpha ⊗F_{weight}+\left(1-\alpha \right)⊗E_{4}\right) \end{array}$$ (6)

where *α* is a learning parameter that allows the model to balance between the original input features and the multi-scale fusion features.

This step makes the module more adaptable in segmentation tasks, especially in polyps with multiple scales and significant contrast variations. Combined with multi-scale and channel attention, the performance of intestinal polyp segmentation was effectively improved.

#### Improved prediction head (PH+)

The overall structure of PH includes the TB and FCB as its input, as illustrated in Fig 1e. Unlike the PH module in FCBFormer [18], we incorporate our DHA module into the PH module, forming PH+. The role of PH+ is to further process the two-branch features for more accurate segmentation prediction, and the addition of DHA can enhance this purpose. By fusing global and local features, the DHA module can intensify the focus on the foreground region, which is crucial for successful segmentation prediction. In conclusion, when integrated into the PH module, the DHA module can leverage the network’s focus on the foreground, thus providing more semantically expressive features for the final segmentation prediction.

#### Dual-channel hybrid attention (DHA)

Recent research shows that attention mechanisms are crucial for enhancing the effectiveness of deep learning models. We propose a new DHA mechanism to better simulate the overall relationship and specific characteristics of the lesion location. This mechanism is based on global context branching and local lesion branching, and it is applied to the PH+ module of our model.

**Fig 3:**
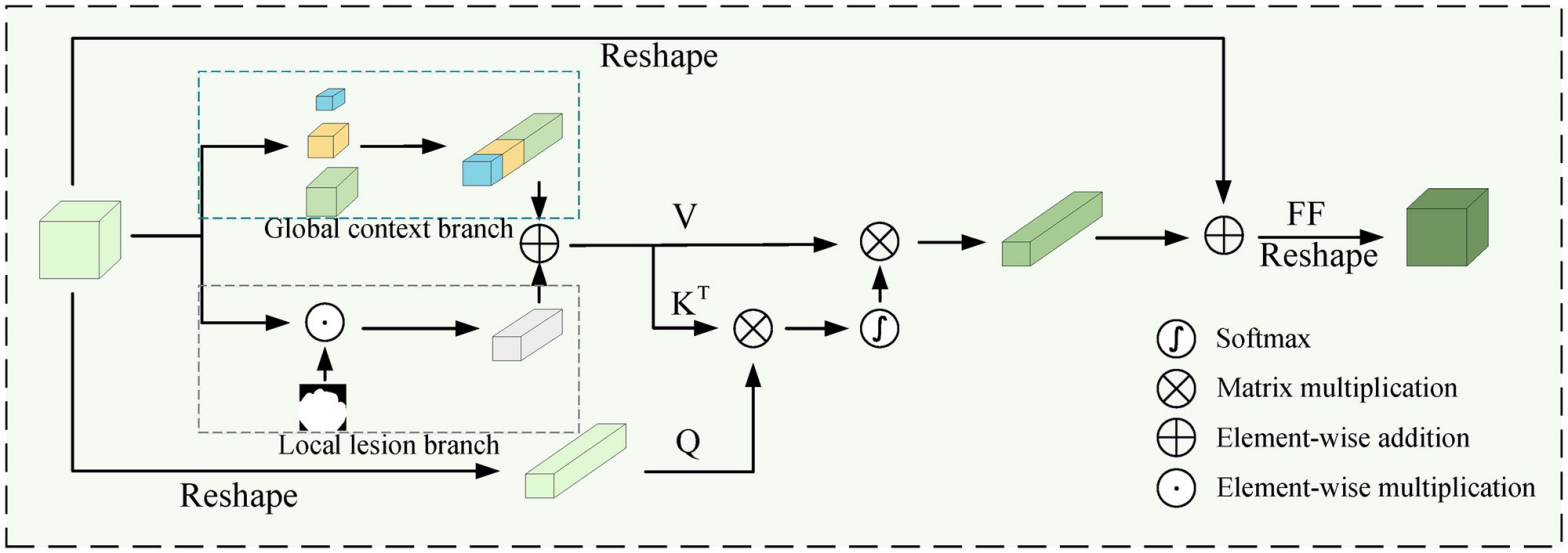
Overall architecture of DHA module.

The DHA module architecture, inspired by transformer components, is depicted in Fig 3. Unlike traditional self-attention, our DHA layer is a novel construct that bifurcates into a global context branch(GCB) and a local lesion branch(LLB). The global context branch utilizes adaptive average pooling with kernel sizes of 1 × 1, 3 × 3, and 5 × 5 to capture multi-scale spatial features from the decoder feature map $D\in R^{C\times H\times W\;}$ creating a global feature representation ${K_{1},V_{1}\in R}^{S\times C}$ by reshaping the pooled outputs and concatenating them (*S* < < *N* and *N* = *H* × *W*). This process effectively expands the receptive field and enhances the feature map with broad contextual information, obtained by a pyramid pooling procedure [42].

For the local lesion branch, an initial segmentation mask $P\in R^{1\times H\times W}$ is applied to *D* using element-wise multiplication, followed by a custom sum pooling operation to distill focused lesion features into *K*_2_ and ${V_{2}\in R}^{1\times C}$. The integration of features from both branches is then performed by summing:

$$\begin{array}{c} K=K_{1}+K_{2},V=V_{1}+V_{2} \end{array}$$ (7)

The output of the DHA layer is formulated as:

$$\begin{array}{c} DHA\left(D\right)=\emptyset_{o}\left(concat\left(head^{0},\ldots ,head^{n}\right)\right) \end{array}$$ (8)

where each *head*^*j*^ represents the output of an individual attention head computed as:

$$\begin{array}{c} head^{j}=Attention\left(\emptyset_{q}^{j}\left(Q\right),\emptyset_{k}^{j}\left(K\right),\emptyset_{v}^{j}\left(V\right)\right) \end{array}$$ (9)

where ∅_*o*_, $\emptyset_{q}^{j}$, $\emptyset_{k}^{j}$, $\emptyset_{v}^{j}$ refers to the linear projection. n denotes the number of multiple heads and the attention formula is as follows:

$$\begin{array}{c} Attention\left(Q,K,V\right)=soft\;\text{max}\left(\frac{QK^{T}}{\sqrt{d_{k}}}\right)V \end{array}$$ (10)

where *d*_*k*_ is the size of each head equivalent to $\frac{C}{n}$.

## Datasets and metrics

### The datasets

Kvasir-SEG [43] and CVC-ClinicDB [44] are two open-access datasets commonly used for gastrointestinal polyp image segmentation. The Kvasir-SEG dataset is one of the datasets that include a sizable number of colonoscopy pictures labeled by medical professionals. This dataset consists of 1000 colonoscopy pictures, along with typical segmentation findings. The labeling and verification of these pictures have been done by knowledgeable gastroenterologists, making it a valuable resource for constructing and evaluating gastrointestinal polyp segmentation algorithms. On the other hand, the CVC-ClinicDB dataset, which consists of 612 images from 29 colonoscopy sequences, is mainly utilized for polyp detection in colonoscopy recordings. Both datasets are open-access and include polyps with various shapes, making them valuable data sources for research in medical image segmentation.

### Evaluation metrics

The tests assess the network performance using the Dice, IoU, Precision, and Recall. the formula is as follows:

$$\begin{array}{c} Dice=\frac{2TP}{2TP+FP+FN} \end{array}$$ (11)

$$\begin{array}{c} IoU=\frac{TP}{TP+FP+FN} \end{array}$$ (12)

$$\begin{array}{c} Precision=\frac{TP}{TP+FP} \end{array}$$ (13)

$$\begin{array}{c} Recall=\frac{TP}{TP+FN} \end{array}$$ (14)

where TP indicates that the classifier predicts a positive result and the sample is actually positive. FP indicates that the classifier predicts a positive result, but the sample is negative. TN denotes that the classifier predicts a negative result and the sample is negative. FN stands for a negative classifier prediction, but the sample’s actual value is positive.

## Results and analysis

### Implementation details

In this study, we created DHAFormer using the PyTorch framework. The network loss function is the Bce loss function and the Dice loss function. The training was conducted with the AdamW optimizer [45], starting with a learning rate of 1e-4. If the performance of the validation set does not improve after 10 cycles, the learning rate is halved. The input resolution was set to 448 × 448, and the batch size was set to 2. We trained DHAFormer for a total of 200 epochs. To adhere to recommendations by [17, 29, 46, 47], an 80%/10%/10% random train/validation/test split was utilized. The data augmentations employed in this study closely resemble those used by the authors of the original FCBFormer [18].

### Comparative experiment

To further illustrate how well the suggested DHAFormer works for segmenting lesions, we also trained and assessed some well-known and cutting-edge instances using the same dataset and assessment measures. These examples included the most sophisticated CNN-based networks, including UNet [10], ResUNet [48], ResUNet++ [46], PraNet [29], HarDNet-MSEG [49] and transformer-based network architectures such as Polyp-PVT [16], SSFormer [17], FCBFormer [18] and DuAT [19]. Meanwhile, to guarantee the impartiality of the experimental comparisons, the same parameter settings and computing environments were used throughout the experimentation, and the findings are displayed in Table 1 for the two datasets. Although some of the earlier models do not perform as well as the latest models in some metrics, they still have some application value.

**Table 1:** Results from our comparative experiments.

Training data	Kvasir-SEG				CVC-ClinicDB					
Metric	Dice(%)	IoU(%)	Prec.(%)	Rec.(%)	Dice(%)	IoU(%)	Prec.(%)	Rec.(%)	Params	FLOPS
UNet [10]	81.47	72.09	85.80	84.31	87.61	79.96	87.49	89.23	31.04M	327.41G
ResUNet [48]	60.34	46.75	58.20	80.54	52.15	39.06	58.39	62.10	0.88 M	42.81G
ResUNet++ [46]	82.99	74.33	88.38	83.23	78.30	66.61	81.92	80.04	4.06 M	94.59G
PraNet [29]	89.93	83.81	90.54	92.54	91.27	84.97	91.45	92.73	30.50 M	41.60G
HarDNet-MSEG [49]	88.31	82.46	89.96	89.04	92.08	87.61	94.72	90.93	17.42 M	36.06G
Polyp-PVT [16]	91.69	86.14	94.35	91.00	93.49	89.17	94.92	92.30	25.11 M	31.7G
SSFormer [17]	91.69	86.69	93.09	92.49	93.48	88.96	94.02	93.30	49.84 M	88.46G
FCBFormer [18]	92.31	87.85	93.93	92.40	93.87	89.26	95.05	93.73	64.52 M	254.14G
DuAT [19]	92.16	87.00	93.33	93.08	94.61	89.88	94.24	95.25	24.95 M	31.30G
Ours	**94.33**	**90.00**	**95.94**	**93.52**	**95.47**	**91.47**	**95.69**	**95.51**	64.90 M	336.89G

Table 1 presents the results of a quantitative comparison of various methods used on the Kvasir-SEG dataset and CVC-ClinicDB dataset and highlights the best results in bold fonts. The results show that our model achieves the best results on both datasets in Dice, IoU, Precision, and Recall. Based on traditional CNN methods, they still perform well. However, our method is much better than the one based on CNN. SSFormer [17] uses the Transformer architecture for global context modeling of image features and enhances the feature representation using spatial concentration and channel attention; FCBFormer [18] is a method based on FCN and transformer, which together perform feature extraction and segmentation of the input image, achieving significant competitive advantages. DHAFormer comprehensively considers the extraction of local features and global features to achieve better segmentation results in polyp segmentation. Compared with other methods, DHAFormer has a larger parameter count and FLOPS, but it has significant advantages in improving segmentation accuracy and recall rate.

**Fig 4:**
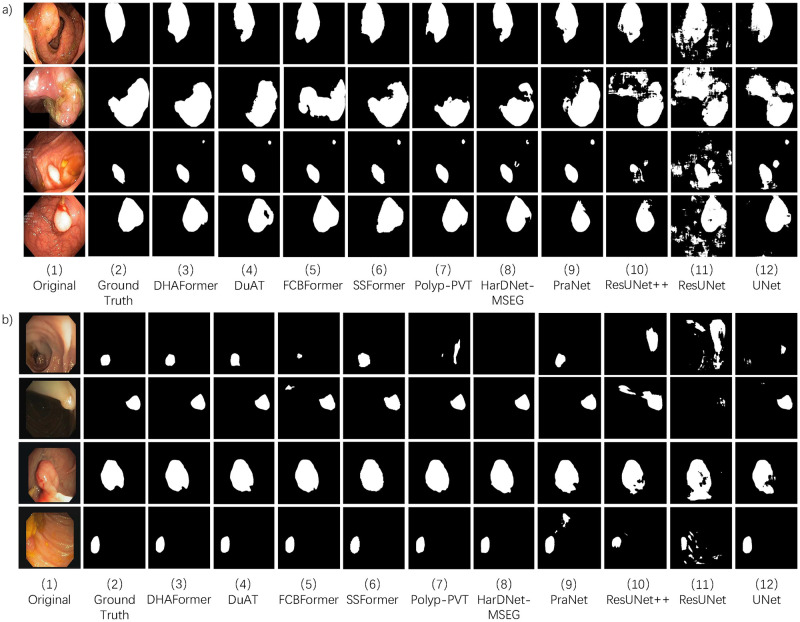
Qualitative comparison results. (a) Qualitative comparison results on Kvasir. (b) Qualitative comparison results on CVC-ClinicDB.

Fig 4a presents qualitative comparisons of the Kvasir-SEG dataset with different approaches. This comparison reveals that the standard convolution method performed poorly in global modeling, making it challenging to identify complicated boundaries in difficult scenarios. The transformer improves these phenomena, however, the transformer-based approach has a weak local modeling capability, as seen from the predicted segmentation maps of SSFormer [17], which has a coarse segmentation profile. As shown in Fig 4, DHAFormer can identify the edge of the polyp more accurately, and its segmentation contour is also smoother and more in line with the growth characteristics of the polyp. The effectiveness of DHAFormer is verified by qualitative analysis.

Fig 4b displays illustrative qualitative results produced using various techniques for some complex cases from the CVC-CliniDB dataset. The DHAFormer effectively measures the relationship between background and foreground information and improves segmentation, as indicated by the first row of qualitative analysis results. The segmentation effect of our proposed network DHAFormer on the boundary of the lesion region is significantly better compared to the current commonly used CNN segmentation networks and more advanced transformer-based methods, as demonstrated in the qualitative analysis results in the second and third rows of Fig 4b. Our method effectively enhances foreground information while suppressing background information, as shown in Fig 4b, verifying the feasibility of DHAFormer for segmentation through comparison with other methods.

### Ablation studies

#### Impact of key components on DHAFormer performance

We conducted ablation tests on the Kvasir-SEG and CVC-ClinicDB datasets and contrasted our model with the baseline (FCBFormer) to more clearly illustrate the impact of each of our parts. The experimental findings are shown in Table 2, demonstrating the importance of the MCFM and DHA modules in this model. We attempted to remove either of these modules during the ablation trial, which resulted in a decrease in network performance. We may, therefore, conclude that adding the MCFM and DHA module is essential for enhancing the functionality of the polyp segmentation model.

**Table 2:** Results from our ablation study.

Dataset	Kvasir-SEG				CVC-ClinicDB			
Metric	Dice(%)	IoU(%)	Prec.(%)	Rec.(%)	Dice(%)	IoU(%)	Prec.(%)	Rec.(%)
Baseline	92.31	87.85	93.93	92.40	93.87	89.26	95.05	93.73
Baseline+MCFM	92.94	88.08	93.69	93.80	94.65	90.19	95.34	94.42
Baseline+DHA	93.23	88.58	93.87	94.38	94.56	90.03	94.97	94.63
Baseline+MCFM+DHA	94.33	90.00	95.94	93.52	95.47	91.47	95.69	95.51

**Fig 5:**
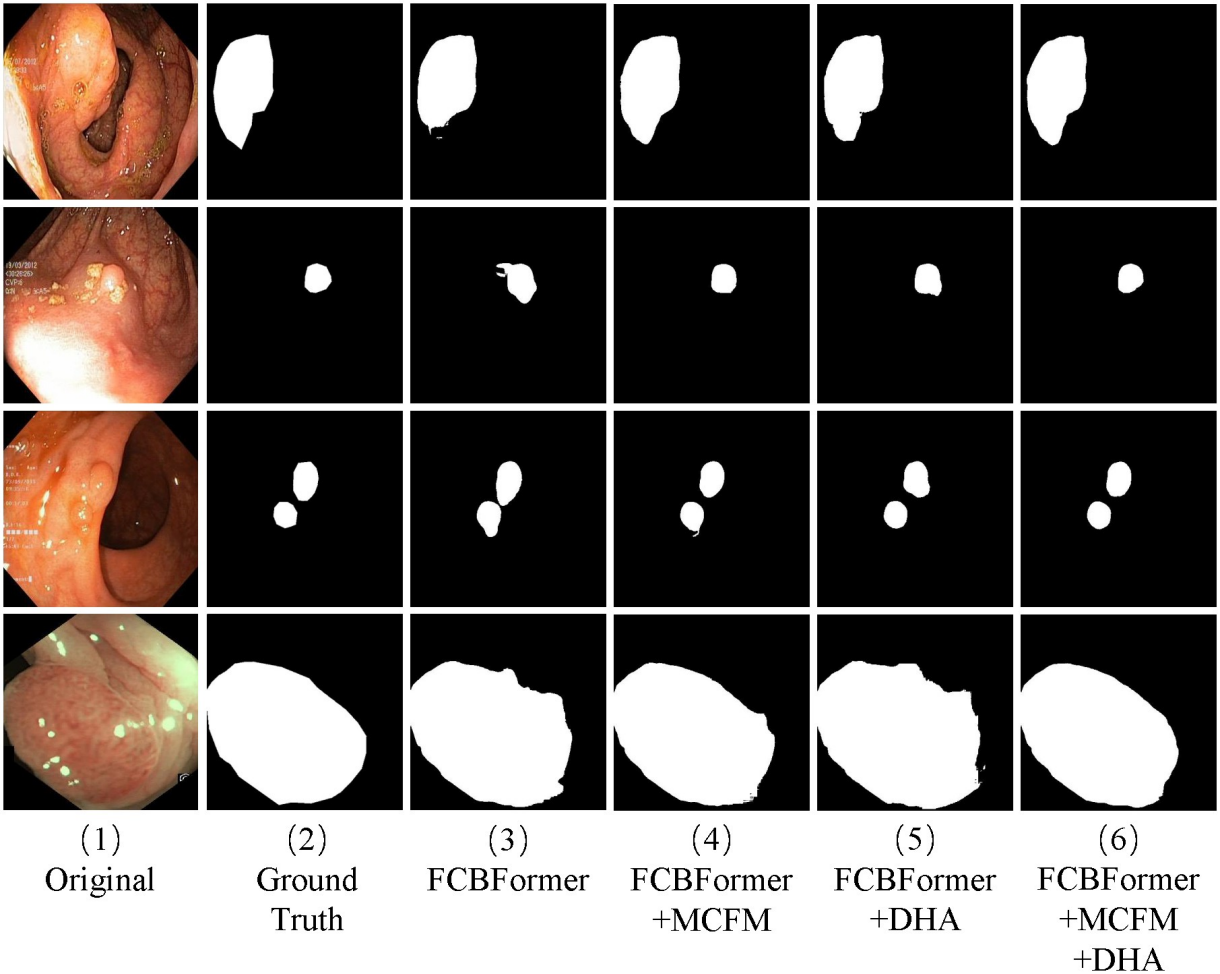
Qualitative results on Kvasir-SEG.

Our DHAFormer method surpasses the Baseline model FCBFormer in all four indices measured on the Kvasir-SEG dataset. Specifically, our method improves the Dice, IoU, Precision, and Recall by 2.02%, 2.15%, 2.01%, and 1.12%, respectively. These results unequivocally establish the superiority of our approach. This improvement in the Dice and IoU indices suggests that the enhanced model effectively captures the foreground information. Furthermore, the improved model demonstrates its ability to better control false alarms and omissions, resulting in improved Precision and Recall indices. It can be seen from Fig 5 that Baseline+MCFM can segment polyp contours more accurately than Baseline. The boundary processing of the foreground part is more accurate and smooth in MCFM, and the segmentation of a large area is closer to the label map. In the first line of Fig 5, it can be observed that the Baseline+DHA enhances the identification of the foreground region of the polyp compared to the Baseline. Combining the MCFM and DHA module enables better capture of foreground information and suppression of background information, resulting in segmentation results that closely align with the labeled image.

**Fig 6:**
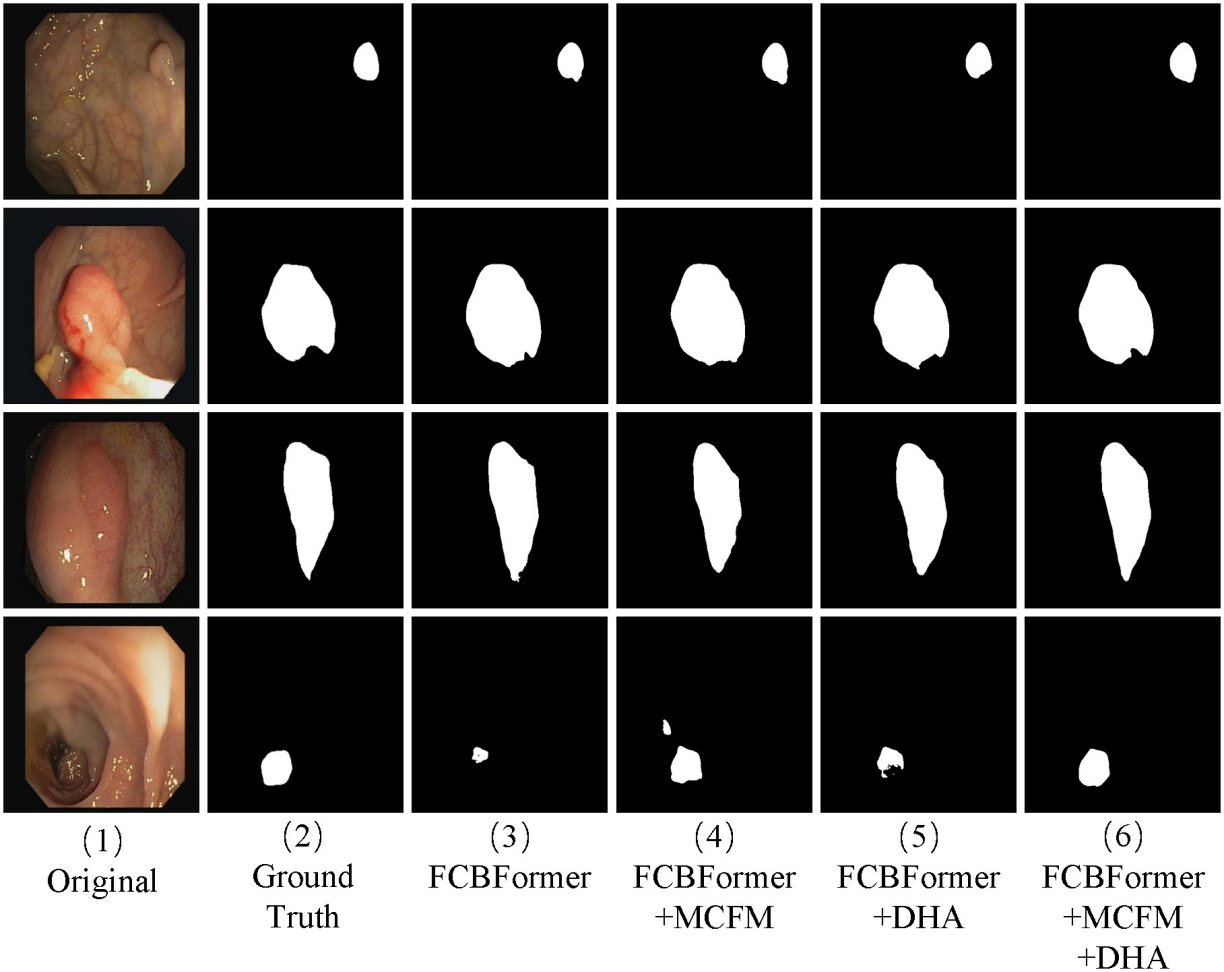
Qualitative results on CVC-ClinicDB.

On the CVC-CliniDB dataset, the segmentation results of Baseline+MCFM+DHA are superior to those of Baseline, Baseline+MCFM, and Baseline+DHA, it is observed that Dice, IoU, Precision, and Recall improve by 1.60%, 2.21%, 0.64%, and 1.78% over the baseline model FCBFormer, respectively. To correctly identify polyp boundary information, the network must focus more on extracting local details. As shown in the fourth line of Fig 6, compared with Baseline, Baseline+MCFM can identify polyp areas more accurately but introduces some redundant information. Baseline+MCFM+DHA can enhance the outlook information and eliminate some redundant information. The combination of MCFM and DHA module can grasp the polyp boundary information more accurately and conduct local modeling.

#### Optimal configuration of MCFM

In order to further explain the importance of the number of convolution operations in MFCM. We did additional ablation experiments, specifically we used three 3 × 3 convolution branches, two 3 × 3 and one 5 × 5 convolution branches, and one 3 × 3, one 5 × 5 and one 7 × 7 convolution branches in MCFM, respectively, to illustrate the effects of varying the number and size of convolution nuclei within MFCM.

In the ablation study in Table 3, DHAFormer model demonstrated significant performance improvement through evaluation of different MCFM configurations. The results showed that the best Dice scores were obtained on the Kvasir-SEG and CVC-ClinicDB data sets using the 3 × 3, 3 × 3 and 5 × 5 convolution combinations, which were 92.94% and 94.65%, respectively. This configuration not only performs the best Dice score and accuracy, but also maintains a reasonable balance in terms of the number of parameters (64.71M) and floating-point arithmetic (258.52G). In contrast, although the 3 × 3, 5 × 5, and 7 × 7 convolution configurations have the highest floating point throughput (262.53G), the performance gains are not significant. Therefore, 3 × 3, 3 × 3, and 5 × 5 convolution combinations are considered to be the most efficient configurations, capable of improving segmentation performance while maintaining high computational efficiency.

**Table 3:** Ablation experiments of different configurations of MCFM.

Dataset	Kvasir-SEG				CVC-ClinicDB					
Metric	Dice(%)	IoU(%)	Prec.(%)	Rec.(%)	Dice(%)	IoU(%)	Prec.(%)	Rec.(%)	Params	FLOPS
Baseline	92.31	87.85	93.93	92.40	93.87	89.26	95.05	93.73	64.52M	254.14G
Baseline+MCFM(3×3+3×3+3×3)	92.77	88.13	94.52	93.10	94.43	89.80	95.39	94.06	64.64M	256.91G
Baseline+MCFM(3×3+5×5+7×7)	92.89	88.06	93.27	94.08	94.05	89.22	94.62	93.95	64.87M	262.53G
Baseline+MCFM(3×3+3×3+5×5)	92.94	88.08	93.69	93.80	94.65	90.19	95.34	94.42	64.71M	258.52G

#### MFCM placement studies

In the internal ablation experiment in Table 4, we evaluated the impact of multi-scale channel fusion module (MCFM) placed at different locations on model performance, demonstrating its important role in multi-level feature fusion. Although adding MCFM at the end of each LE module has the highest Dice score of 93.01%, this configuration is higher on floating-point arithmetic (271.52G), resulting in increased computing costs. We finally chose to add MCFM after the fourth LE module and achieved a Dice score of 92.94%, which maintained a reasonable balance in terms of the number of parameters (64.71M) and floating point arithmetic (258.52G). Therefore, we chose to add MCFM after the fourth LE module in order to maintain high computational efficiency while guaranteeing high performance.

**Table 4:** Ablation experiments of MCFM at different locations on the Kvasir-SEG dataset.

Network	MCFM Location	Dice(%)	IoU(%)	Prec.(%)	Rec.(%)	Params	FLOPS
Baseline	After 1st LE	92.52	87.73	93.26	93.48	64.71M	258.52G
	After 2nd LE	92.58	87.70	94.21	92.90	64.71M	258.52G
	After 3rd LE	92.74	88.04	94.80	92.87	64.71M	258.52G
	After 4th LE	92.94	88.08	93.69	93.80	64.71M	258.52G
	After every LE	93.01	88.29	94.19	93.46	64.71M	271.52G

#### DHA internal ablation experiment

We configured both GCB and LLB in the DHA module. To isolate the effects of each component of DHA, we conducted ablation experiments inside DHA that will make their individual contributions to model performance clearer.

In the internal ablation experiment in Table 5, the DHAFormer model demonstrated significant performance improvement by evaluating the independent contribution of the GCB and the LLB. The results showed that the combination of GCB and LLB configurations achieved the highest Dice scores on the Kvasir-SEG and CVC-ClinicDB data sets, which were 93.23% and 94.56%, respectively. This configuration not only performs well in Dice score and accuracy, but also maintains a reasonable balance in terms of the number of parameters (64.72M) and floating-point arithmetic (332.56G). In contrast, a configuration using only GCB or LLB, while also improving, is not as significant as a combination of the two. Therefore, the configuration combining GCB and LLB is considered to be the most efficient and can improve segmentation performance while maintaining high computational efficiency.

**Table 5:** Results of DHA internal ablation experiment.

Dataset	Kvasir-SEG				CVC-ClinicDB					
Metric	Dice(%)	IoU(%)	Prec.(%)	Rec.(%)	Dice(%)	IoU(%)	Prec.(%)	Rec.(%)	Params	FLOPS
Baseline	92.31	87.85	93.93	92.40	93.87	89.26	95.05	93.73	64.52M	251.14G
Baseline+GCB	92.94	87.98	93.49	93.98	94.41	89.59	95.51	93.66	64.72M	331.35G
Baseline+LLB	92.67	87.73	93.33	93.99	94.24	89.55	94.71	94.32	64.72M	331.36G
Baseline+GCB+LLB	93.23	88.58	93.87	94.38	94.56	90.03	94.97	94.63	64.72M	332.56G

#### Generalizability tests

We conducted a generalization test of DHAFormer, following the conventions outlined by [17, 18, 47]. Specifically, in this test, we assessed the performance of the model trained in Kvasir-SEG on CVC-ClinicDB and vice versa. The results of these generalizability tests, which can be found in Table 6, indicate that DHAFormer excelled in processing images with slightly different distributions compared to the training dataset. Notably, it outperformed existing models in most metrics.

**Table 6:** Results from our generalisability tests.

Training data	Kvasir-SEG				CVC-ClinicDB			
Test data	CVC-ClinicDB				Kvasir-SEG			
Metric	Dice(%)	IoU(%)	Prec.(%)	Rec.(%)	Dice(%)	IoU(%)	Prec.(%)	Rec.(%)
UNet [10]	76.31	66.86	89.70	72.04	46.37	34.08	40.40	86.19
ResUNet [48]	61.04	47.45	75.95	59.87	35.16	24.02	26.80	87.85
ResUNet++ [46]	77.74	67.71	81.28	79.29	45.12	33.01	45.94	65.30
PraNet [29]	86.78	79.99	84.74	**92.91**	68.88	59.08	65.92	**89.92**
HarDNet-MSEG [49]	88.31	82.46	89.96	89.04	79.55	70.75	86.24	81.72
Polyp-PVT [16]	89.61	83.35	91.36	89.82	81.79	74.40	93.41	77.94
SSFormer [17]	91.04	85.08	92.76	91.78	85.39	79.10	**94.97**	82.52
FCBFormer [18]	90.94	85.20	90.36	92.71	87.38	80.72	92.18	87.08
DuAT [19]	89.45	82.65	89.06	91.89	87.73	80.96	93.34	86.46
Ours	**91.42**	**85.67**	**94.21**	90.80	**87.80**	**81.91**	92.71	86.18

## Conclusion

In this study, we introduce a dual-channel hybrid attention network with transformer (DHAFormer). This novel polyp segmentation architecture adopts a multi-scale channel fusion module (MCFM) and dual-channel hybrid attention (DHA) for dense prediction. Our goal is to improve the model’s ability to identify and segment polyps accurately. On the one hand, MCFM combines multi-scale features and channel attention to increase the sensitivity of the network to polyp size. On the other hand, the DHA module simulates both global and local features to enhance the network’s attention to the foreground polyp area. This enhancement enables the model to efficiently identify and segment hidden polyp areas that are easily overlooked. The combination of the MCFM and DHA module demonstrated superior performance compared to the baseline model, as evidenced by improvements in Dice, IoU, Precision, and Recall metrics. This underscores the effectiveness of our proposed DHAFormer network for lesion segmentation. In future work, we aim to optimize the network for efficiency while improving our understanding of the network’s local data.

While our proposed DHAFormer model demonstrates superior performance in polyp segmentation tasks, it has a higher number of parameters and FLOPs compared to some state-of-the-art methods. This increased complexity could impact its applicability in real-time or resource-constrained environments. We acknowledge this limitation and will address it in our future research by optimizing the model to reduce its computational demands while maintaining high segmentation accuracy, making it more suitable for practical applications.
